# Supplementary material for: Metabolomic Aspects of Conservative and Resistance-Related Elements of Response to Fusarium culmorum in the Grass Family
Source: Cells. 2022 Oct 13;11(20):3213. doi: 10.3390/cells11203213 (PMC9600661; doi:10.3390/cells11203213)
Supplement: Supplementary file 1 [file cells-11-03213-s001.zip › Supplementary method 1.pdf]

Supplementary Methods

To better characterised *Fusarium culmorum* isolate KF846, the gene expression of key biosynthetic genes (ZEA synthase – ZEA2, Zinc finger transcription factor - TRI6, Trichodiene synthase - TRI5) were performed.

The fungal mycelium for RNA extraction was cultured *in vitro* in 50 ml Czapek-Dox broth (Sigma-Aldrich) with Yeast Extract (Oxoid) and streptomycin sulphate (50 mg/L) for 5 days at 25°C with rotary shaking at 100 rpm. The samples were collected every 24 h. RNA was extracted and purified using RNeasy Plant Mini Kit (Qiagen, Hilden, Germany) according to the manufacturers' protocol with the additional DNase digestion step. The quality of total RNA was estimated by Nanodrop (Thermo Scientific, Wilmington, NC, USA). Real-time RT-PCR reactions were performed using an CFX96 Touch™ Real-Time PCR Detection System (Bio-Rad, Hercules, CA). Analyses were conducted using iTaq One Step SYBR Green RT-qPCR Kit (Bio-Rad, Hercules, California, USA). The total reaction volume was 20 µL: 10 µL iTaq One Step SYBR Green RT-qPCR mix, 2 µL RNA (< 30 ng), 0.5 µL each primer (10 µM), 0.125 µL reverse transcriptase and 6.875 µL nuclease free water. The reaction was carried out using the following protocol: initial denaturation 94 °C for 2 min, followed by 40 cycles at 94 °C for 15 s, 59 °C for 1 min. In the experiment, three biological and two technical replicates were performed. Primers used for *ZEA2, TRI5, TRI6,* β-tubulin gene expression analysis were as follow: rtZEA2_em_fA3 GGT GGA CAC TTC TTG AAG CA; rtZEA2_em_rA1 CAG TGG TAG TAC CAG CAA CCT; rtTRI5_em_fA4 ACT TAC AGT CCA TAG TGC CTA CG; rtTRI5_em_rA4 CTC CAA AGA GTG CAT GGC GGA T; rtTRI6_em-fA1 CAA GCC AGC TCA TCG CCC T; rtTRI6_em-rA1 TGT TGT CGG TAA TGC CGC CT; BtubF GCC TCG ACA GCA ATG GTG TT; BtubR CCG GAC TGA CCG AAA ACG AA [1]. Relative quantification of gene expression was calculated using the $2^{-\Delta\Delta Ct}$ method (Bio-Rad, Hercules, CA) with β-tubulin as endogenous control.

Quantification of Fusarium culmorum

Used primers according to [2]:

MGB-F TCACCCAAGACGGGAATGA

MGB-R GAACGCTGCCCTCAAGCTT

MGB probe CACTTGGATATATTTCC

1. Dawidziuk, A.; Koczyk, G.; Popiel, D. Adaptation and response to mycotoxin presence in pathogen-pathogen interactions within the Fusarium genus. *World Mycotoxin Journal* **2016**, *9*, 565-575, doi:10.3920/wmj2015.2010.
2. Waalwijk, C.; van der Heide, R.; de Vries, I.; van der Lee, T.; Schoen, C.; Costrel-de Corainville, G.; Häuser-Hahn, I.; Kastelein, P.; Köhl, J.; Lonnet, P.; et al. Quantitative Detection of Fusarium Species in Wheat Using TaqMan. *European Journal of Plant Pathology* **2004**, *110*, 481-494, doi:10.1023/B:EJPP.0000032387.52385.13.
