# Supplementary figures and images for: Metabolomic Aspects of Conservative and Resistance-Related Elements of Response to Fusarium culmorum in the Grass Family
Source: Cells. 2022 Oct 13;11(20):3213. doi: 10.3390/cells11203213 (PMC9600661; doi:10.3390/cells11203213)

RT: 0.00 - 20.00 SM: 15G

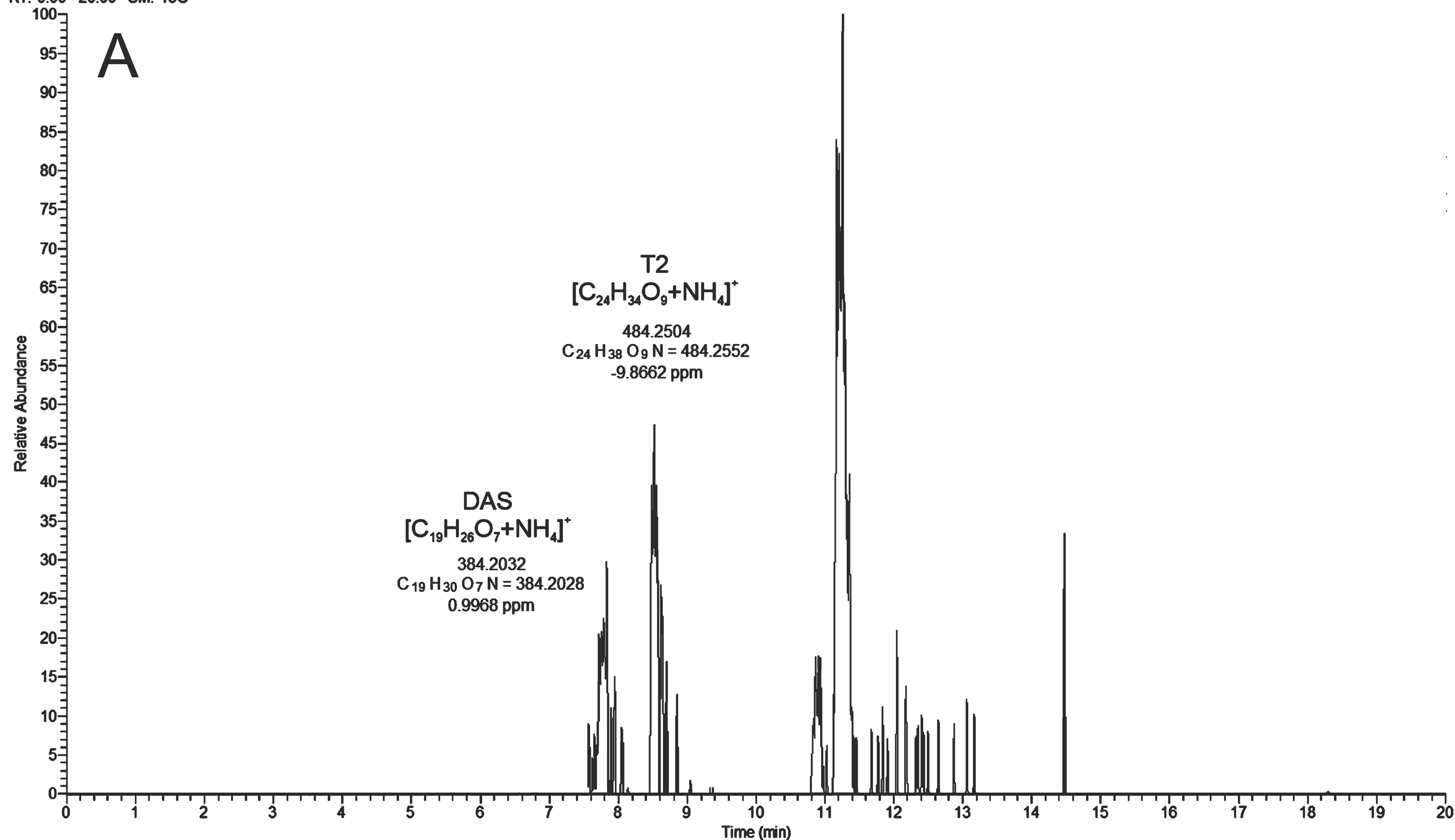

**DON**  
 $[C_{15}H_{20}O_6+CH_3COO]^-$   
355.1396  
 $C_{17}H_{23}O_8 = 355.1398$   
-0.7138 ppm

RT: 0.00 - 20.01 SM: 15G

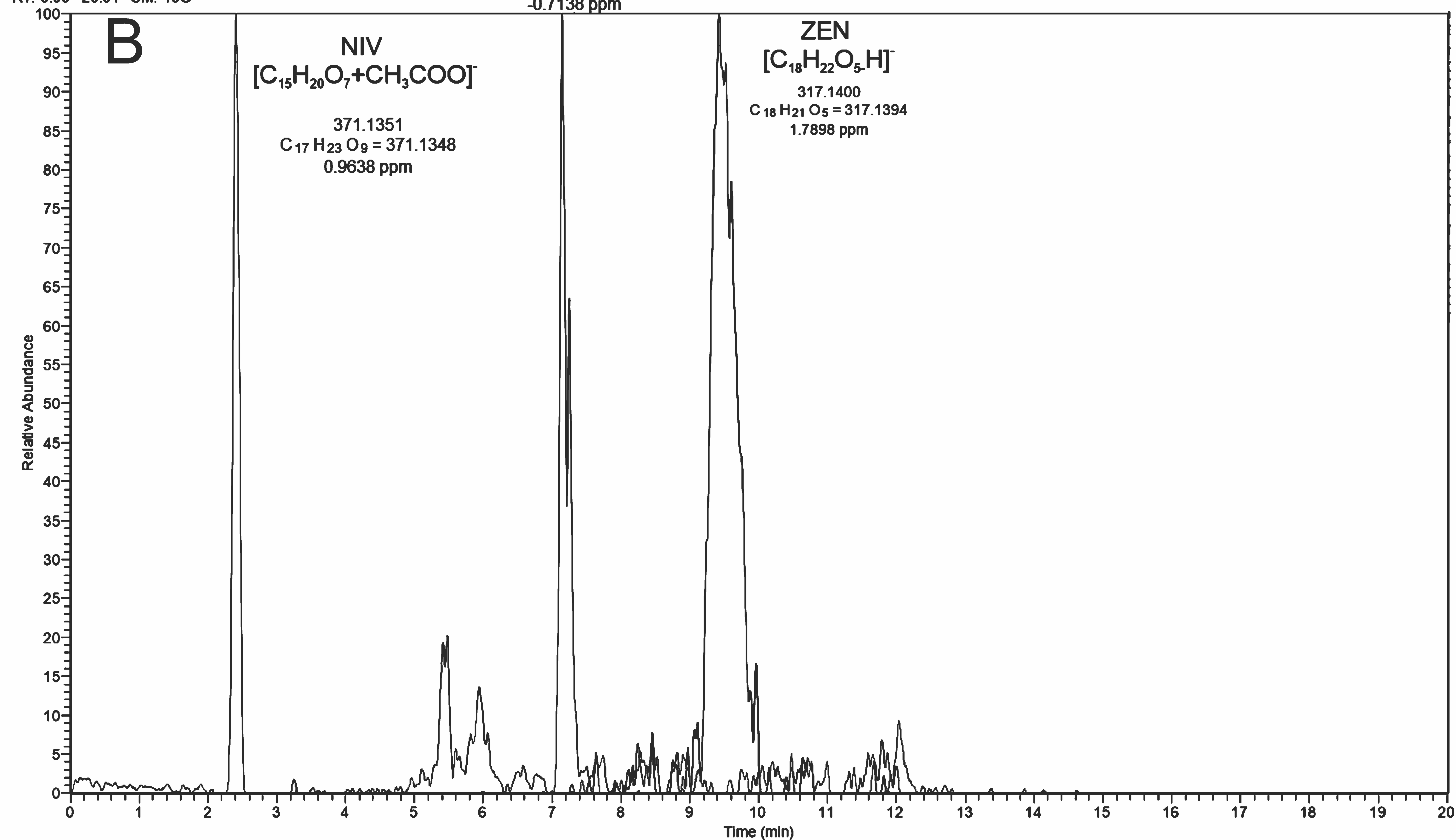

Supplement: Supplementary file 1 [file cells-11-03213-s001.zip › Figure S1.pdf]

# B

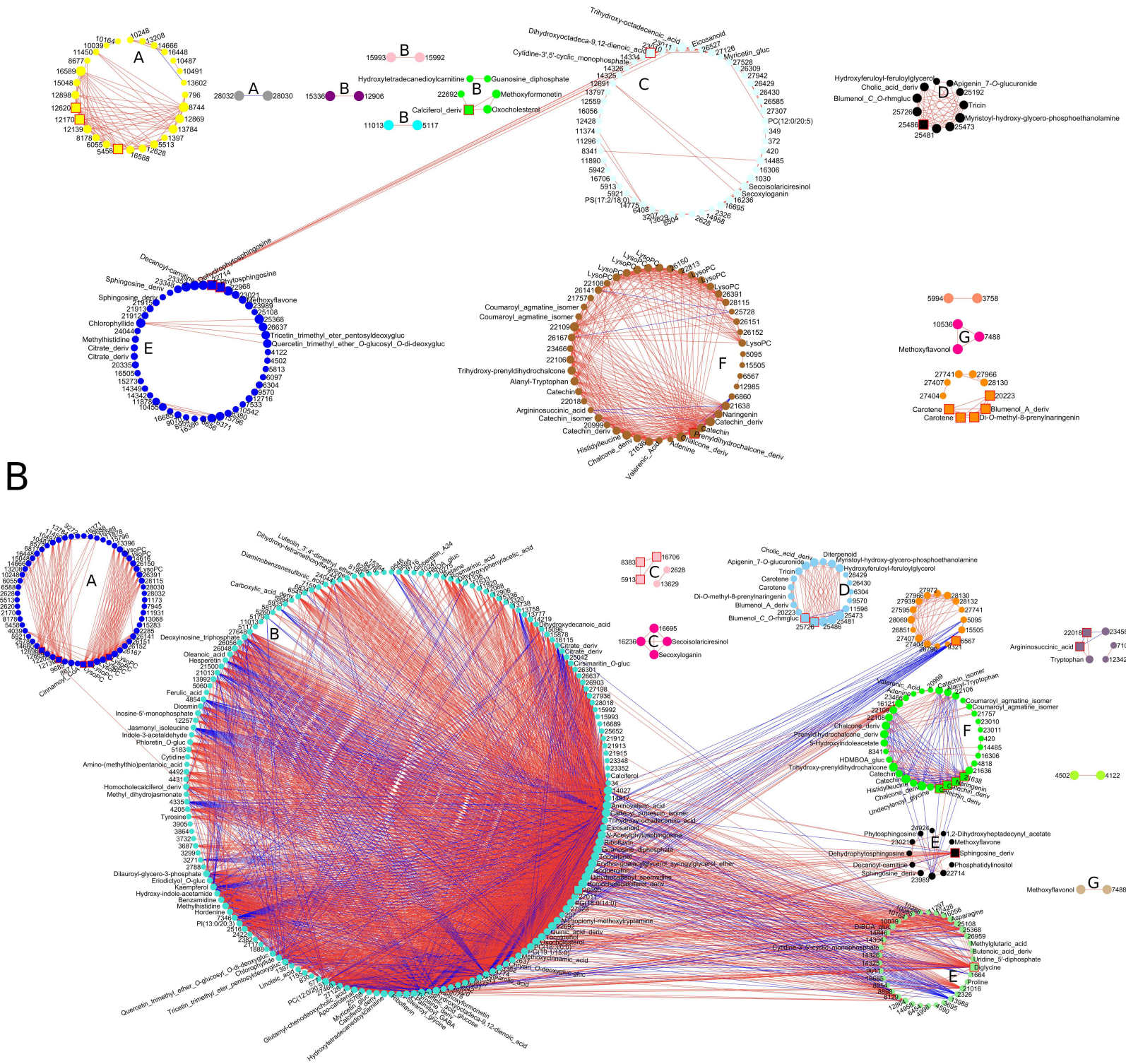

Supplement: Supplementary file 1 [file cells-11-03213-s001.zip › Figure S2.pdf]
